# On the Mechanical Friction Losses Occurring in Automotive Differential Gearboxes

**DOI:** 10.1155/2014/523281

**Published:** 2014-01-19

**Authors:** Grégory Antoni

**Affiliations:** Haute Ecole d'Ingénierie et de Gestion du Canton de Vaud, Institut COMATEC, Route de Cheseaux 1, 1401 Yverdon-les-Bains, Switzerland

## Abstract

In the automobile industry, the mechanical losses resulting from friction are largely responsible for various kinds of surface damage, such as the scuffing occurring in some mechanical assemblies. These scuffing processes seem to be due to a local loss of lubrication between certain mechanical elements of the same assembly, leading to a sharp increase in the friction, which can lead to a surface and volume damage in some of them, and even can cause, in the worst case, the whole destruction of the mechanical system if it has continued to operate. Predicting and checking the occurrence of this kind of undesirable phenomena, especially in some principal systems of the vehicle, represents nowadays, a crucial challenge in terms of automobile reliability and safety. This study focuses on the mechanical friction losses liable to occur in differential automobile gearboxes, which can lead in the long term to the scuffing of these mechanical systems. The friction losses involved were modeled, using a simple analytical approach, which is presented and discussed.

## 1. Introduction

Although the automobile industry has contributed significantly during the last few years to increasing the CO_2_ levels polluting the atmosphere, the introduction of a Carbon Tax has been inciting car manufacturers to reduce this pollution. In line with this more environmentally friendly approach, automobile manufacturers are now attempting to decrease the mechanical friction losses occurring between some vehicle parts—causing both an increase in fuel consumption and levels of carbon dioxide emissions—while maintaining their company's competitiveness on the market. In practice, these friction losses are frequently associated with surface damage of several kinds such as scuffing [1–6] which is also known as galling [7] or seizure [8–10]. Scuffing is an extreme form of adhesive wear which occurs when two mechanical parts adhere locally to each other and a material transfer takes place from the one surface to the other during a sliding process, which in turn can result in the welding or binding of the entire mechanical system [11–14]. Although the physical causes of scuffing have not yet been clearly established, it is generally recognized that they are initiated when the lubricant film between two sliding parts is suddenly destroyed as the result of a sharp temperature increase due to the presence of heavy mechanical loads [15]. The most accurate and efficient methods of predicting scuffing processes are those based on estimating the thermal energy responsible for this damage in comparison with that produced under normal operating conditions [16, 17]. The friction energy dissipated in the mechanical assemblies in question must therefore thus be assessed to define a criterion in order to predict the occurrence of the resulting scuffing effects [16, 17]. The mechanical losses to which the differential gearboxes are subject, can lead to a surface and volume damage of one or more elements of this system, and causing, in the worst case, the blocking and the destruction of the system, if it remains in operation. In this study on the mechanical friction losses occurring in differential automobile gearboxes, an analytical model was developed (see Section 2) in order to determine the contribution of each mechanical component to the overall friction losses. In Section 3, the relative contribution of each of the constitutive components of the model and the effects of various parameters on the mechanical friction losses occurring in a differential gearbox are discussed.

## 2. Analysis of the Mechanical Friction Losses Occurring in Differential Automobile Gearboxes: Constitutive Equations

### 2.1. General Description

**Figure 1:**
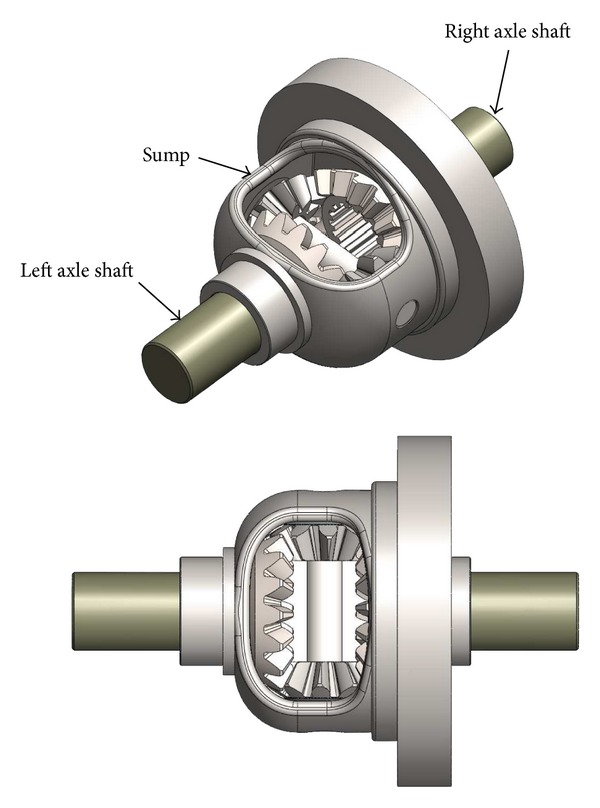
Differential gearbox (with its sump and the axle shafts).

**Figure 2:**
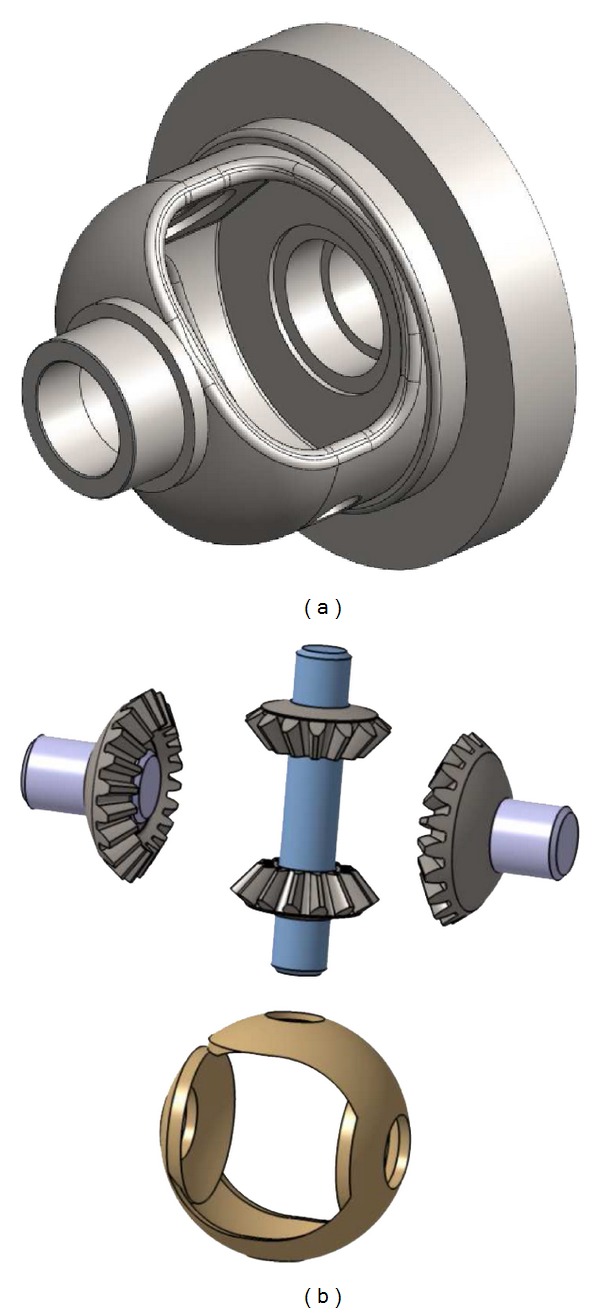
(a) Sump of a differential gearbox. (b) Various components of a differential mechanism.

**Figure 3:**
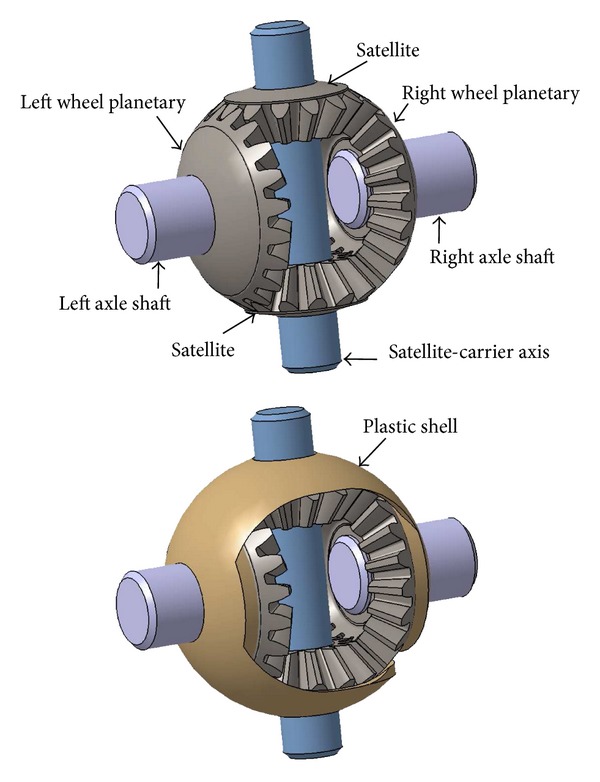
Components of a differential gearbox: two planetary bevel pinions, two satellite bevel pinions with their shaft and the plastic shell.

Generally speaking, a differential gearbox [18] (Figure 1) is a complex device involving gears which are connected to output shafts transmitting both rotation and torque forces. In the framework of motor vehicles, differential systems of this kind make it possible to deliver an equal or different speed to all the wheels, depending on whether the vehicle is taking a straight or curvilinear course: when the vehicle is starting to turn, the inner wheels must rotate more slowly than those in the outside because they have to cover a smaller distance during the same time span. Differential systems consist of four bevel gears (two “planetary” pinions and two “satellite” pinions), a satellite-carrier axis, and a plastic shell (Figure 2); all these components are enclosed in a nonsymmetric sump entrained by the differential ring gear, which is itself driven in a rotating manner by both the primary and secondary gearbox shafts, which are made to rotate by the engine of the vehicle. The two planetary bevel pinions connected to each axle shaft placed opposite each other are meshed by the two smaller satellite pinions (Figure 3). When the vehicle is moving straight ahead, the two planetary pinions rotate at the same speed (as does the axle shaft associated with each of the driven wheel) and the two satellite pinions are stationary relative to their axis; that is, the differential housing and these pinions rotate at the same speed as the ring gear. However, when the vehicle turns, the rotational speed of the wheel (and that of the corresponding planetary pinions) differs from that of the two satellite pinions, which start rotating around their axis in order to compensate for this difference.

### 2.2. Power and Yield Equations

The total gearbox yield, *η*_gb_, is obtained by dividing the sum of the output powers of each of the driven wheels by the gearbox's input power:

(1) $$\begin{array}{c} \eta_{\text{gb}}=\frac{P_{\text{lw}}+P_{\text{rw}}}{P_{\text{en}}}=\frac{C_{\text{lw}}\omega_{\text{lw}}+C_{\text{rw}}\omega_{\text{rw}}}{C_{\text{en}}\omega_{\text{en}}}, \end{array}$$

where *P*_*i*_, *C*_*i*_, and *ω*_*i*_ (with *i* = (lw, rw, en)) are powers, torques, and angular velocities associated with the left wheel, right wheel, and engine, respectively.

Upon introducing both the primary and secondary gearbox shaft yields, the ratio between the power of the differential ring gear and that of the engine is equal to the product of these shaft yields; that is,

(2) $$\begin{array}{c} \eta_{\text{ps}}\eta_{\text{ss}}=\frac{P_{\text{rg}}}{P_{\text{en}}}=\frac{C_{\text{rg}}\omega_{\text{rg}}}{C_{\text{en}}\omega_{\text{en}}}, \end{array}$$

where *η*_ps_ and *η*_ss_ denote the primary and secondary shaft yields, respectively, and *P*_rg_, *C*_rg_ and *ω*_rg_ are the power, torque and angular velocity associated with the differential ring gear (drive gear), respectively.

On the other hand, the total gearbox yield can be written as the product of the primary and secondary shaft yields and the differential gearbox:

(3) $$\begin{array}{c} \eta_{\text{gb}}=\eta_{\text{ps}}\eta_{\text{ss}}\eta_{\text{di}}. \end{array}$$

After combining (1)–(3), the total differential gearbox yield, *η*_di_, can be obtained by dividing the sum of the output powers of each of the driven wheels by the input power:

(4) $$\begin{array}{c} \eta_{\text{di}}=\frac{P_{\text{lw}}+P_{\text{rw}}}{P_{\text{rg}}}=\frac{C_{\text{lw}}\omega_{\text{lw}}+C_{\text{rw}}\omega_{\text{rw}}}{C_{\text{rg}}\omega_{\text{rg}}}. \end{array}$$

Since a differential gearbox can both transmit and distribute the differential ring gear power (the input power) to the output gears associated with each of the driven wheels, it follows that

(5) $$\begin{array}{c} P_{\text{rg}}=P_{\text{lw}}+P_{\text{rw}}+P_{\text{ab}}, \end{array}$$

where *P*_ab_ is the power absorbed in the differential gearbox due to the friction losses.

**Figure 4:**
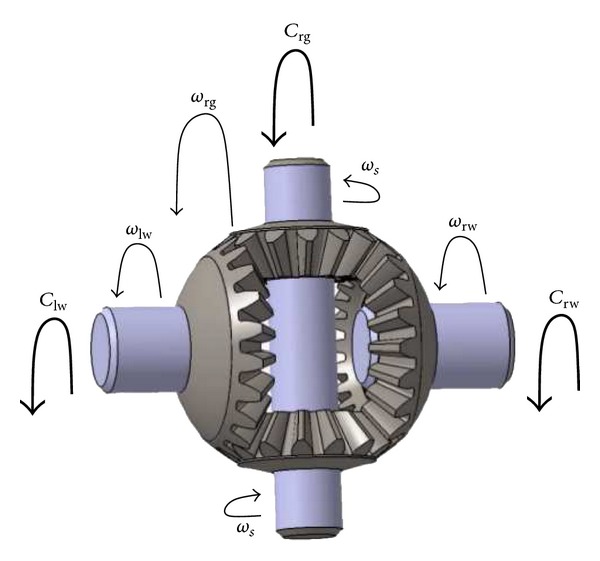
Angular velocities and torques in the differential gearbox.

In the equations governing the angular velocities and the torques in the differential gearbox (Figure 4),

(6a) $$\begin{array}{c} \omega_{\text{lw}}R_{p}=\omega_{\text{rg}}R_{p}-\omega_{s}R_{s}, \end{array}$$

(6b) $$\begin{array}{c} \omega_{\text{rw}}R_{p}=\omega_{\text{rg}}R_{p}+\omega_{s}R_{s}, \end{array}$$

(7) $$\begin{array}{c} C_{\text{rg}}=C_{\text{lw}}+C_{\text{rw}}, \end{array}$$

where *ω*_*s*_ denotes the angular velocity of satellite pinion and *R*_*p*_ and *R*_*s*_ are the pitch radii of planetary and satellite pinions, respectively.

Adding or subtracting (6a)-(6b), we obtain the classical relations:

(8a) $$\begin{array}{c} \omega_{\text{rg}}=\frac{\omega_{\text{lw}}+\omega_{\text{rw}}}{2}, \end{array}$$

(8b) $$\begin{array}{c} \omega_{s}=\frac{\omega_{\text{rw}}-\omega_{\text{lw}}}{2}\frac{R_{p}}{R_{s}}. \end{array}$$

Note that from now on, Δ*ω* = |*ω*_lw_ − *ω*_rw_ | ≥0 and Δ*C* = |*C*_rw_ − *C*_lw_ | ≥0 will be used to denote the difference in the angular velocity and torque between the driven wheels, respectively (where |·| is the absolute-value function).

Combining (5) and (6a), (6b), and (7), the power absorbed in the differential can be written as

(9) $$\begin{array}{c} P_{\text{ab}}=\frac{1}{2}\Delta C\Delta \omega . \end{array}$$

Based on the above equations, the total differential gearbox yield is

(10) $$\begin{array}{c} \eta_{\text{di}}=1-\frac{P_{\text{ab}}}{P_{\text{rg}}}=1-\left(\frac{\Delta C\Delta \omega }{2C_{\text{rg}}\omega_{\text{rg}}}\right). \end{array}$$

*Comments*

- Under straight driving conditions, the angular velocities of two satellite pinions in relation to the satellite axis are zero (Δ*ω* = 0) and the speed of the driven wheels is equal to the angular velocity of the differential ring gear; that is, *ω*_lw_ = *ω*_rw_ = *ω*_rg_.
- In curvilinear driving situations, the angular velocity of two satellite pinions in relation to the satellite-carrier axis is no longer zero (Δ*ω* > 0) and the speeds of the driven wheels are no longer equal; that is, *ω*_lw_ > *ω*_rw_ (right turn) or *ω*_lw_ < *ω*_rw_ (left turn).
- If one of the driven wheels undergoes slipping [18]—if it is the left (resp., right) wheel, *ω*_lw_ = 0 (resp., *ω*_rw_ = 0), that is, Δ*ω* = 2*ω*_rg_—the differential gearbox yield reduces to *η*_di_ = 1 − (Δ*C*/*C*_rg_).

### 2.3. Mechanical Losses: Friction Torques

The power absorbed in the differential gearbox is the sum of the power dissipated at the various contact points existing in the overall mechanism, which can be decomposed as follows:

(11) $$\begin{array}{c} P_{\text{ab}}=P_{\text{sa/pl}}+P_{\text{ax/sa}}+P_{\text{sa/sh}}+P_{\text{pl/sh}}, \end{array}$$

where *P*_sa/pl_, *P*_ax/sa_, *P*_sa/sh_, and *P*_pl/sh_ correspond to the part of the power dissipated between the two satellite and planetary bevel pinions, the satellite pinion and the satellite-carrier axis, between the two bevel heads of the satellite pinions and the surrounding plastic shell, and the two bevel heads of the planetary pinions and the surrounding plastic shell (see Figure 3).

#### 2.3.1. Power Dissipated between Two Satellite Bevel Pinions and Two Planetary Bevel Pinions

In order to account for the power dissipated between the two satellite bevel pinions and the two planetary bevel pinions in the differential mechanism (giving four meshing contacts), we introduce a yield, *η*_sa/pl_ (denoting the power transmitted in the gear between satellite/planetary pinions) which can be written as follows:

(12) $$\begin{array}{c} \eta_{\text{sa/pl}}=\frac{P_{\text{rg}}-P_{\text{sa/pl}}}{P_{\text{rg}}}H^{+}\left(\Delta \omega \right), \end{array}$$

where *P*_sa/pl_ denotes the power dissipated between the two satellite/planetary contacts and *H* ^+^(·) is a Heaviside-like function (adapted in order to ensure that *H* ^+^(*x*) = 1 when *x* > 0 and *H*(*x*) = 0 when *x* ≤ 0). Note that a yield *η*_sa/pl_, with a nonzero value (i.e., *η*_sa/pl_ > 0), occurs between the two satellite pinions and two planetary pinions only when the angular velocity differs between the driven wheels; that is, Δ*ω* > 0.

Based on (12), we can therefore write

(13) $$\begin{array}{c} P_{\text{sa/pl}}=\left(1-\eta_{\text{sa/pl}}\right)C_{\text{rg}}\omega_{\text{rg}}H^{+}\left(\Delta \omega \right). \end{array}$$

#### 2.3.2. Power Dissipated between the Satellite Bevel Pinion and Satellite-Carrier Axis

**Figure 5:**
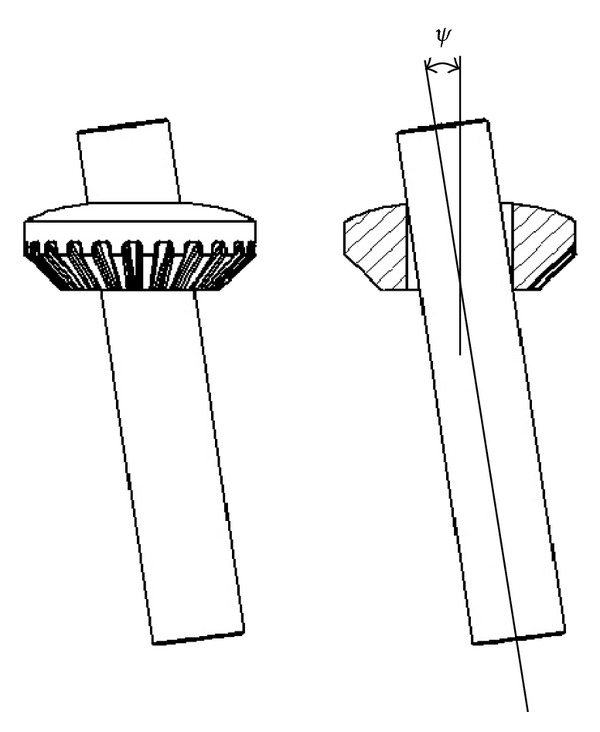
Over-centre mechanism between the satellite pinion and satellite-carrier axis; *ψ* denotes the angular eccentricity.

Neglecting the presence of an over-centre mechanism between the satellite pinion and satellite-carrier axis (Figure 5), the force exerted on the satellite-carrier axis, *F*_ax_, upon the rotation of the differential ring gear (*ω*_rg_) can be written as

(14) $$\begin{array}{c} F_{\text{ax}}=\frac{C_{\text{rg}}}{2R_{p}}. \end{array}$$

In (14), the point of application of the force, *F*_ax_, is assumed to take the same contact path as both the left and right meshing gears (Figure 7).

**Figure 6:**
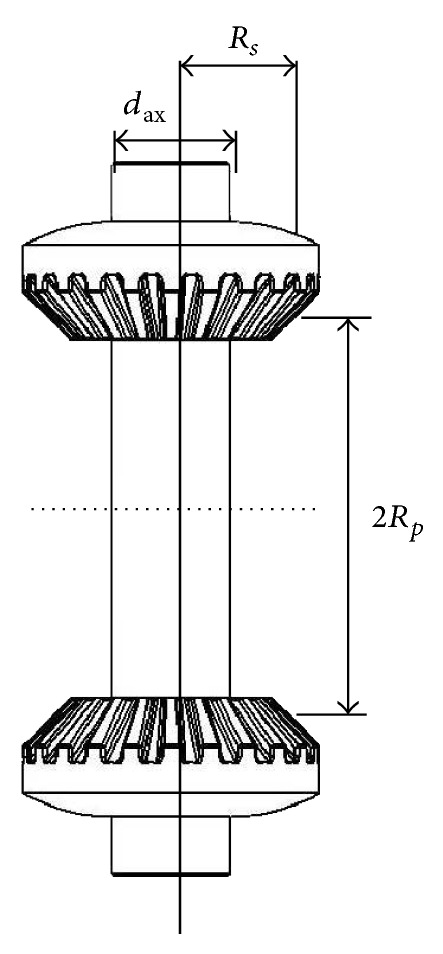
Satellite bevel pinions and satellite-carrier axis.

Adopting a Coulomb-type friction law, the friction torque applied to the satellite-carrier axis, *C*_ax/sa_^*f*^, is

(15) $$\begin{array}{c} C_{\text{ax/sa}}^{f}=\mu_{\text{ax/sa}}F_{\text{ax}}\frac{d_{\text{ax}}}{2}, \end{array}$$

where *μ*_ax/sa_ denotes the friction coefficient (which is constant) between the satellite pinion and the satellite-carrier axis and *d*_ax_ is the axis diameter (Figure 6).

Combining (14) and (15), the power dissipated between these two assembly components reads

(16) $$\begin{array}{c} P_{\text{ax/sa}}=2C_{\text{ax/sa}}^{f}\omega_{s}=\mu_{\text{ax/sa}}\frac{C_{\text{rg}}}{4}\frac{d_{\text{ax}}}{R_{s}}\Delta \omega . \end{array}$$

#### 2.3.3. Power Dissipated between the Satellite Bevel Pinions and the Plastic Shell

The power dissipated between the two bevel satellite pinions and the surrounding plastic shell, *P*_sa/sh_, can be written as

(17) $$\begin{array}{c} P_{\text{sa/sh}}=2C_{\text{sa/sh}}^{f}\omega_{s}=C_{\text{sa/sh}}^{f}\frac{R_{p}}{R_{s}}\Delta \omega , \end{array}$$

where *C*_sa/sh_^*f*^ denotes the friction torque applied to the satellite bevel pinion. Note that the friction torque, *C*_sa/sh_^*f*^, is assumed here to be identical on the two satellite pinions.

In order to write the equations giving the equilibrium of each satellite pinion, which is meshed with the two planetary pinions associated with the driven wheels and in contact with the satellite-carrier axis as well as with the plastic shell, we use the Fundamental Principle of Statics:

(18) $$\begin{array}{c} {\left\{T_{\text{lw}\to S}\right\}}_{M}+{\left\{T_{\text{rw}\to S}\right\}}_{M}+{\left\{T_{\text{ax}\to S}\right\}}_{M}+{\left\{T_{\text{sh}\to S}\right\}}_{M}=\left\{0\right\}, \end{array}$$

where {*T*_*i*→*S*_}_*M*_ denotes the transmittable force torsor of solid *i* (and *i* = (lw, rw, ax, sh) associated with the planetary pinions of the left and right wheels, the satellite-carrier axis, and the plastic shell, resp.) exerted on solid *S* (the satellite pinion) at point *M* (see [15]).

**Figure 7:**
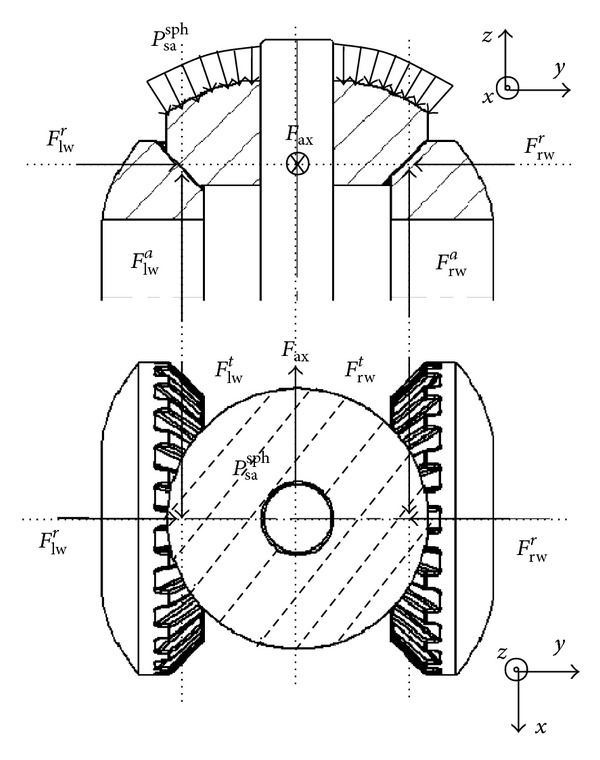
Forces applied to a satellite pinion.

Looking only at the equilibrium of the resulting force of each torsor in the direction(*x*, *y*, *z*) (see Figure 7), (18) writes

**Figure 8:**
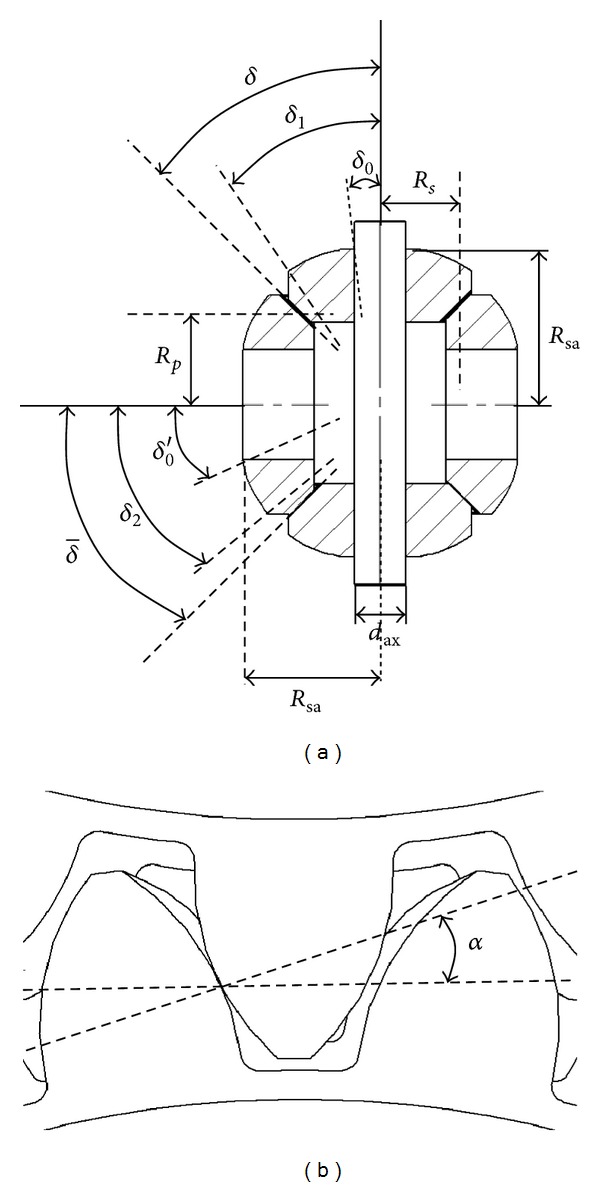
Geometry of a differential gearbox.

(19)  {FlwtFlwrFlwa+{Frwt−FrwrFrwa+{−Fax00+{00−Xsa={000

(20) ⟺{Flwt+Frwt=FaxFlwr=Frwr(Flwt+Frwt)tanαsinδ=Xsa,

where *F*_*j*_^*t*^, *F*_*j*_^*r*^, and *F*_*j*_^*a*^ (with *j* = lw, rw) are the tangential, radial, and axial forces of *j*-planetary pinions (associated with the left and right wheels, Figure 7; see [19]), respectively, *X*_sa_ is the resulting force applied to the satellite pinion generated by the plastic shell, *δ* is the half-angle pitch radius of the satellite pinion (see Figure 8(a)), and *α* is the pressure angle between the satellite and planetary pinions, which is assumed here to be identical between the satellite/planetary meshing gears (see Figure 8(b)).

**Figure 9:**
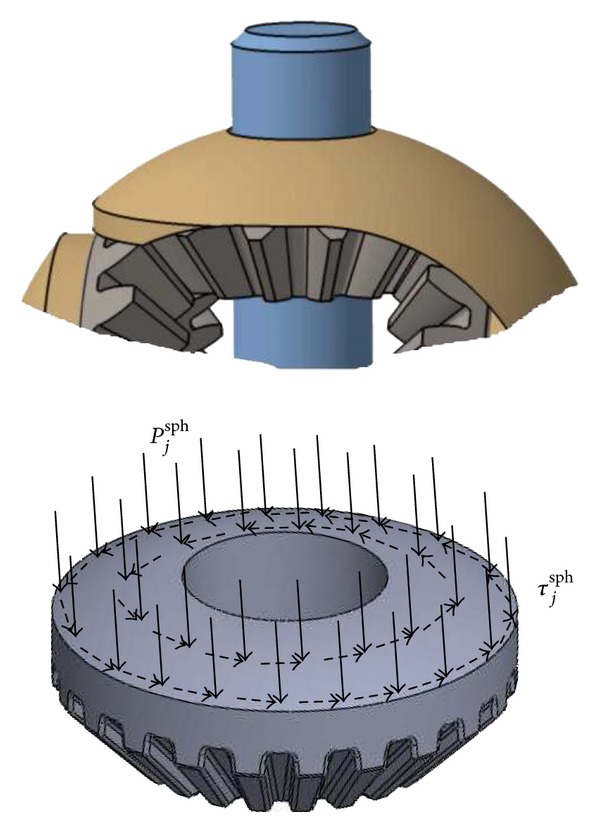
Pressure *P*_*j*_^sph^ (solid arrow) and shear *τ*_*j*_^sph^ (dashed arrow) fields on a head-pinion *j* (with *j* = (sa, pl)) caused by the plastic shell.

The pressure applied to the head surface of the satellite pinion *P*_sa_^sph^ (its spherical part, see Figure 9) due to the resulting force, *X*_sa_ (in line with (20)) matches the relationship (see [15]):

(21) $$\begin{array}{c} X_{\text{sa}}=\overset{2\pi }{\underset{0}{\int }}\overset{\delta_{1}}{\underset{\delta_{0}}{\int }}P_{\text{sa}}^{\text{sph}}\left(\theta ,\phi \right)R_{\text{sa}}^{2}\cos\theta \sin\theta \;d\theta \;d\phi , \end{array}$$

where *δ*_1_ and *δ*_0_ are the angles defining the spherical part of the head-satellite pinion and *R*_sa_ is the radius of the inner sphere of the plastic shell (where the contact with the head surface of the satellite pinion and the plastic shell occurs).

The expression for the friction torque *C*_sa/sh_^*f*^ applied to the head surface of the satellite pinion can be written (see [17]) as

(22) Csa/shf=∫02π∫δ0δ1τsasph(θ,ϕ)Rsa3sinθ dθ dϕ=∫02π∫δ0δ1μsa/shPsasph(θ,ϕ)Rsa3sinθ dθ dϕ,

where *τ*_sa_^sph^(*θ*, *ϕ*) = *μ*_sa/sh_ *P*_sa_^sph^(*θ*, *ϕ*) denotes the shear stress exerted on the head surface of the satellite pinion since a Coulomb-type friction law is adopted (see Figure 9) and *μ*_sa/sh_ is the coefficient of friction (which is constant) between the satellite pinion and the plastic shell.

Combining (14) and (20)–(22) and assuming that, in a first approximation, the pressure field exerted on the head-satellite pinion is uniform (i.e., *P*_sa_^sph^(*θ*, *ϕ*) = *P*_sa_^sph^), (21) and (22) reduce to

(23)  Xsa  =2πRsa2Psasph(cos⁡(2δ0)−cos⁡(2δ1)4),Csa/shf=μsa/shRsaCrg2Rptanαsinδ×(cos⁡(δ1+δ0)sin(δ1−δ0)−(δ1−δ0)sin(δ0+δ1)sin(δ0−δ1)).

#### 2.3.4. Power Dissipated between the Planetary Bevel Pinions and the Plastic Shell

The power dissipated between the two planetary pinions and the plastic shell, *P*_pl/sh_, can be written as

(24) $$\begin{array}{c} P_{\text{pl/sh}}=C_{\text{pl/sh}}^{f(\text{lw})}\omega_{\text{lw}}^{\text{rs}}+C_{\text{pl/sh}}^{f(\text{rw})}\omega_{\text{rw}}^{\text{rs}}, \end{array}$$

where *C*_pl/sh_^*f*(*i*)^ are the frictional torque between the planetary *i*-pinion (*i* = (lw, rw) refers to the left wheel (lw) and the right wheel (rw)) and the plastic shell, and *ω*_*i*_^rs^ = Δ*ω*/2 denotes the relative speed of the *i*-pinion in relation to the sump differential.

Assuming that *C*_pl/sh_^*f*(lw)^≅*C*_pl/sh_^*f*(rw)^ = *C*_pl/sh_^*f*^, (24) reads

(25) $$\begin{array}{c} P_{\text{pl/sh}}=C_{\text{pl/sh}}^{f}\Delta \omega . \end{array}$$

Using the same procedure as above, namely, meshing each of the planetary pinions with two satellite pinions, which means that the resulting force applied to the head-planetary pinion by the plastic shell, *X*_pl_, is

(26) $$\begin{array}{c} X_{\text{pl}}=F_{\text{ax}}\tan\alpha \sin\overset{-}{\delta }, \end{array}$$

where $\overset{-}{\delta }$ is the half-angle pitch radius of the planetary pinion (see Figure 8(a)). Note that given the particular geometry of the mechanism, the sum of the half-angle pitch radii of the planetary and satellite pinions satisfies $\delta +\overset{-}{\delta }=\pi /2$, and we therefore obtain the following relationship: $\sin\overset{-}{\delta }=\cos\delta$.

The expressions for both the resulting force, *X*_pl_, and the friction torque, *C*_pl/sh_^*f*^, applied to the head-satellite pinion are:

(27) $$\begin{array}{c} X_{\text{pl}}=\overset{2\pi }{\underset{0}{\int }}\overset{\delta_{2}}{\underset{\delta_{0}^{\prime }}{\int }}P_{\text{pl}}^{\text{sph}}\left(\theta ,\phi \right)R_{\text{sa}}^{2}\cos\theta \sin\theta \;d\theta \;d\phi ,\\ C_{\text{pl/sh}}^{f}=\overset{2\pi }{\underset{0}{\int }}\overset{\delta_{2}}{\underset{\delta_{0}^{\prime }}{\int }}\tau_{\text{pl}}^{\text{sph}}\left(\theta ,\phi \right)R_{\text{sa}}^{3}\sin\theta \;d\theta \;d\phi , \end{array}$$

where *τ*_pl_^sph^(*θ*, *ϕ*) = *μ*_pl/sh_ *P*_pl_^sph^(*θ*, *ϕ*) denotes the shear stress exerted on the outer surface of the planetary pinion with a Coulomb-type friction law (Figure 9), *μ*_pl/sh_ is the coefficient of friction (which is constant) between the satellite pinion and the plastic shell, *P*_pl_^sph^ is the pressure applied to the head surface of the planetary pinion, and *δ*_2_ and *δ*_0_′ are the angles defining the spherical portion of the head-planetary pinion.

It is again assumed here that in a first approximation, the pressure field, *P*_pl_^sph^, exerted on the head-planetary pinion is uniform (i.e., (*θ*, *ϕ*)-independent), and (27) reduce to

(28) Xpl=2πRsa2Pplsph(cos⁡(2δ0′)−cos⁡(2δ2)4),Cpl/shf=μpl/shRsaCrg2Rptanαcos⁡δ×(cos⁡(δ2+δ0′)sin(δ2−δ0′)−(δ2−δ0′)sin(δ0′+δ2)sin(δ0′−δ2)).

#### 2.3.5. Total Power Dissipated in the Differential Gearbox

Based on the above constitutive equations, the total power dissipated in the differential gearbox can be written as follows:

(29) Pab=Psa/pl+Pax/sa+Psa/sh+Ppl/sh=(1−ηsa/pl)CrgωrgH+(Δω)+μax/saCrg4daxRsΔω +μsa/shRsaCrg2Rstanαsinδ ×(cos⁡(δ1+δ0)sin(δ1−δ0)−(δ1−δ0)sin(δ0+δ1)sin(δ0−δ1))Δω +μpl/shRsaCrg2Rptanαcos⁡δ ×(cos⁡(δ2+δ0′)sin(δ2−δ0′)−(δ2−δ0′)sin(δ0′+δ2)sin(δ0′−δ2))Δω.

Using (10) and (29) and assuming that in a first approximation, *μ*_sa/sh_ = *μ*_pl/sh_ = *μ*_sh_ (where *μ*_sh_ denotes the coefficient of friction between each of the pinions in contact with the plastic shell), the total differential gearbox yield can be written as follows:

(30) ηdi=1−∑ili   =1−[Psa/plPrg︸=lsa/pl+Pax/saPrg︸=lax/sa+Psa/shPrg︸=lsa/sh+Ppl/shPrg︸=lpl/sh],

with

(31) lsa/pl=(1−ηsa/pl)H+(Δω),lax/sa=μax/sadax4RsΔωωrg,lsa/sh=μshRsa2Rstanαsinδ×(cos⁡(δ1+δ0)sin(δ1−δ0)−(δ1−δ0)sin(δ0+δ1)sin(δ0−δ1))Δωωrg,lpl/sh=μshRsa2Rptanαcos⁡δ×(cos⁡(δ2+δ0′)sin(δ2−δ0′)−(δ2−δ0′)sin(δ0′+δ2)sin(δ0′−δ2))Δωωrg,

where *l*_*i*_ = *P*_*i*_/*P*_rg_ denotes the loss ratio of the *i*-mechanism (with *i* = (sa/pl, ax/sa, sa/sh, pl/sh)). It is worth noting that the differential yield, *η*_di_, shows mechanical friction losses whenever the rotational-speed differs between two of the planetary pinions associated with the driven wheels, that is, when Δ*ω* > 0.

## 3. Discussions 

In the first part of this section, we discuss the order of magnitude of the constitutive parameters of the model presented in Section 2. In the second part, we present a sensitivity analysis in which the response of the model to a given load parameter was examined in order to assess the effect of this response on the mechanical losses occurring in the differential gearbox mechanism.

### 3.1. Order of Magnitude of the Constitutive Parameters

This analytical model involves thirteen parameters (*η*_sa/pl_, *μ*_ax/sa_, *μ*_sh_, *d*_ax_, *R*_*s*_, *R*_*p*_, *R*_sa_, *α*, *δ*, *δ*_0_, *δ*_0_′, *δ*_1_, *δ*_2_) consisting of (i) ten geometrical parameters (*d*_ax_, *R*_*s*_, *R*_*p*_, *R*_sa_, *α*, *δ*, *δ*_0_, *δ*_0_′, *δ*_1_, *δ*_2_) depending on the differential gearbox under consideration, which can be identified fairly easily; (ii) one yield parameter (*η*_sa/pl_) corresponding to the power transmitted by the gear between the satellite/planetary pinions; (iii) two friction parameters (*μ*_ax/sa_, *μ*_sh_) which depend considerably on the types of materials in contact and the surface conditions, their state of lubrication, and their temperature.

Concerning (i), although these specific parameters depend on the differential gearbox under consideration, their order of magnitude can be said to be *d*_ax_ ≈ 10–20 mm, *R*_*s*_ ≈ 20–30 mm, *R*_*p*_ ≈ 20–30 mm, *R*_sa_ ≈ 30–50 mm, and for the angle parameters: *δ*_0_ ≈ 10°–15°, *δ*_0_′ ≈ 15°–25°, *δ*_1_ ≈ 30°–40°, *δ*_2_ ≈ 35°–45°, *α* ≈ 20–25°, *δ* ≈ 30°–40°; readers can refer to Fanchon [19] for further details about some of them.

Concerning (ii), the parameter *η*_sa/pl_, which denotes the power transmitted by the gear between satellite/planetary pinions using a yield term, has a value ranging between 0.92 and 0.98. Since Fanchon [19] has reported that the yield in the case of two meshed pinions is around 98%, it can be concluded that in a differential mechanism, where there are four pinions in contact, the lowest value of *η*_sa/pl_ ≈ (0.98)^4^≅0.92 and the highest value is likely to be *η*_sa/pl_≅0.98. It therefore seems reasonable to assume that the real yield *η*_sa/pl_ of this complex mechanism is approximately 0.96.

The parameters that need to be investigated more closely in order to determine their influence on the mechanical losses are the various coefficients of friction (iii): *μ*_ax/sa_ and *μ*_sh_. The sliding contacts in the differential gearbox mechanism can be of very different kinds [20, 21], since both the satellite/planetary bevel pinion and satellite-pinion/satellite-carrier axis in contact are in the mixed elastohydrodynamic lubrication (MEHL) mode [22–24], which is a mixed regime between (1) elastohydrodynamic lubrication (EHL) [25, 26], where the heavy loads exerted at the surface of the two gears (due to the presence of both high contact pressures and strong transmitted torques) induce elastic strains in the two solids in contact with thin lubricant films, and (2) boundary lubrication (BL) [27], which may tend to constitute a completely dry frictional contact [28], leading to the development of scuffing effects [29, 30]. The contacts between the bevel head pinion (satellites and planetaries) and the plastic shell are in the elastohydrodynamic lubrication mode (EHL) which can lead to the development of the hydrodynamic lubrication regime (HL) [31, 32], where the lubricant film thickness is large enough to completely separate the directly apposed surfaces so that the loads applied between the surfaces are restored and balanced under specific operating conditions. Under the above conditions, the contacts existing between the satellite pinions and the satellite-carrier axis are of the steel/steel contact with lubrication type, *μ*_ax/sa_ ∈ [0.05,0.07], and those existing between the head pinions and the plastic shell are rather of the steel/Teflon contact with lubrication type, *μ*_sh_ ∈ [0.03,0.05].

### 3.2. Sensitivity Analysis: Assessment of the Model's Response to a Given Load Parameter and Prediction of the Mechanical Losses

In what follows, it is proposed to test the model's response to a given load parameter and to determine the influence of some parameters (*η*_sa/pl_, *μ*_ax/sa_, *μ*_sh_) on the mechanical losses occurring in a differential gearbox mechanism. The expression for the load parameter Δ*ω*/*ω*_rg_ ∈ [0,2] means that (i) when Δ*ω*/*ω*_rg_ = 0 (in cases where *ω*_lw_ ≠ 0 and *ω*_rw_ ≠ 0), the vehicle is travelling on a straight line: *η*_di_ = 1 and *l*_*i*_ = *P*_*i*_/*P*_rg_ = 0, ∀*i*; (ii) when 0 < Δ*ω*/*ω*_rg_ < 2, the vehicle is taking a curvilinear course: *η*_di_ < 1 and *l*_*i*_ = *P*_*i*_/*P*_rg_ > 0, ∀*i*; (iii) when Δ*ω*/*ω*_rg_ = 2, one of the driven wheels undergoes slipping (the extreme situation), the vehicle is stationary: *η*_di_ < 1 and *l*_*i*_ = *P*_*i*_/*P*_rg_ > 0, ∀*i*.

The values adopted for the other parameters are *d*_ax_ = 18 × 10^−3^ m, *R*_*s*_ = 22 × 10^−3^ m, *R*_*p*_ = 27 × 10^−3^ m, *R*_sa_ = 49 × 10^−3^ m, *δ*_0_ = 10°, *δ*_0_′ = 18°, *δ*_1_ = 32°, *δ*_2_ = 40°, *α* = 20°, *δ* = 39°.

**Figure 10:**
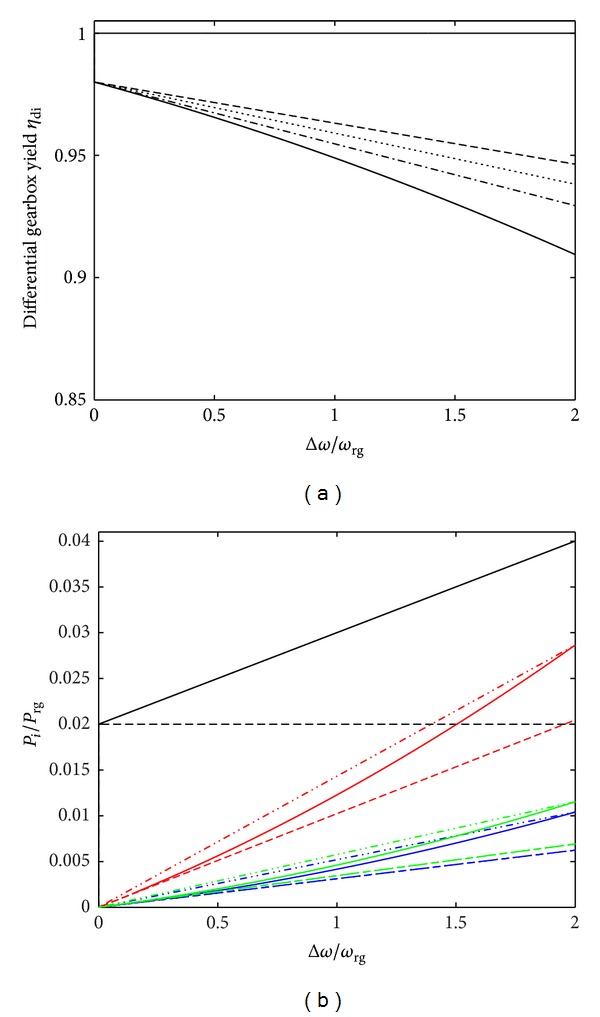
(a) Yield of a differential gearbox *η*_di_ as a function of the load parameter Δ*ω*/*ω*_rg_ for various sets of coefficients of friction (*μ*_ax/sa_, *μ*_sh_): (0.03, 0.05) in a dashed line, (0.05, 0.07) in a dash-dot line, (0.03, 0.07) in a dotted line and in a solid line for (*μ*_ax/sa_, *μ*_sh_), and *η*_sa/pl_ undergoing a concomitant linear increase from (0.03, 0.05) to (0.05, 0.07) and 0.98 to 0.96, respectively, with Δ*ω*/*ω*_rg_; the thick solid line gives the yield of the differential gearbox without any losses (which is constant) with the load parameter Δ*ω*/*ω*_rg_; that is, *l*_*i*_ = *P*_*i*_/*P*_rg_ = 0, ∀*i*. (b) Loss ratio *l*_*i*_ = *P*_*i*_/*P*_rg_ of the *i*-mechanism (with *i* = (sa/pl, ax/sa, sa/sh, pl/sh)) as a function of the load parameter Δ*ω*/*ω*_rg_ and with the same sets of coefficients of friction as in (a): *l*_sa/pl_ without (black solid line) or with (black dashed line) a linear decrease in *η*_sa/pl_ from 0.98 to 0.96, *l*_ax/sa_ (red line), *l*_sa/sh_ (blue line), and *l*_pl/sh_ (green line).

**Figure 11:**
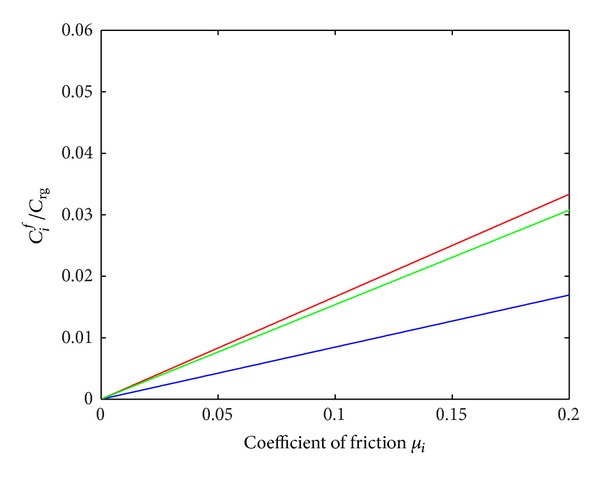
Friction torque ratio *C*_*i*_/*C*_rg_ as a function of coefficient of friction *μ*_*i*_ with *i* = (ax/sa, sa/sh, pl/sh) (while the other parameters were taken to be constant): *C*_ax/sa_/*C*_rg_ increased linearly with *μ*_ax/sa_ (red solid line), *C*_sa/sh_/*C*_rg_ increased linearly with *μ*_sa/sh_ = *μ*_sh_ (blue solid line), *C*_pl/sh_/*C*_rg_ increased linearly with *μ*_pl/sh_ = *μ*_sh_ (green solid line) in a range of variation *μ*_*i*_ ∈ [0,0.2].

Figure 10(a) shows the yield of a differential gearbox *η*_di_ depending on the load parameter Δ*ω*/*ω*_rg_ with various sets of friction coefficients of friction (*μ*_ax/sa_, *μ*_sh_) and a linear decrease in *η*_sa/pl_ from 0.98 to 0.96. In the most gentle operating range, that is, 0 ≤ Δ*ω*/*ω*_rg_ ≤ 1, *η*_di_ decreased from 0.98 to around 0.963, (*μ*_ax/sa_, *μ*_sh_) = (0.03,0.05), *η*_di_ ∈ [0.98,0.959] (resp., *η*_di_ ∈ [0.98,0.954]) when (*μ*_ax/sa_, *μ*_sh_) = (0.03,0.05) (resp., (*μ*_ax/sa_, *μ*_sh_) = (0.05,0.07)) and *η*_di_ decrease from 0.98 to around 0.949 when (*μ*_ax/sa_, *μ*_sh_) showed a concomitant linear increase from (0.03,0.05) to (0.05,0.07). In the heavy operating range, that is, 1 < Δ*ω*/*ω*_rg_ ≤ 2, the maximum differential gearbox yield *η*_di_ ∈ ]0.963,0.946] was obtained with (*μ*_ax/sa_, *μ*_sh_) = (0.03,0.05), and the minimum yield (*η*_di_ ∈ ]0.949,0.909]) occurred when (*μ*_ax/sa_, *μ*_sh_) and *η*_sa/pl_ increased linearly from (0.03,0.05) to (0.05,0.07) concomitantly with the load parameter Δ*ω*/*ω*_rg_ *η*_di_ ∈ ]0.959,0.938] (resp., *η*_di_ ∈ ]0.954,0.929]) when (*μ*_ax/sa_, *μ*_sh_) = (0.03,0.05) (resp., (*μ*_ax/sa_, *μ*_sh_) = (0.05,0.07)). The results presented in Figure 10(b) show the loss ratio *l*_*i*_ = *P*_*i*_/*P*_rg_ associated with *i*-mechanism (where *i* = (sa/pl, ax/sa, sa/sh, pl/sh)) in decreasing order of influence: *l*_ax/sa_ (red line), *l*_sa/pl_ (black line), *l*_pl/sh_ (green line), *l*_sa/sh_ (blue line) when *η*_sa/pl_ had a constant value of 0.98, and in the case where *η*_sa/pl_ decreased from 0.98 to 0.96, the effects of the loss ratio *l*_sa/pl_ became greater than those of the other parameters (*l*_ax/sa_, *l*_ax/sa_, *l*_sa/sh_, *l*_pl/sh_). It should be noted that when 0 ≤ Δ*ω*/*ω*_rg_ ≤ 1, then *l*_ax/sa_ ∈ [0,0.0143], *l*_pl/sh_ ∈ [0,0.0057], and *l*_sa/sh_ ∈ [0,0.0052], whereas when 1 < Δ*ω*/*ω*_rg_ ≤ 2, then *l*_ax/sa_ ∈ [0,0.0286], *l*_pl/sh_ ∈ [0,0.0115], and *l*_sa/sh_ ∈ [0,0.0104].  Figure 11 shows the friction torque ratio *C*_*i*_/*C*_rg_ as a function of the friction coefficient *μ*_*i*_ with *i* = (ax/sa, sa/sh, pl/sh) when the other parameters were taken to be fixed constants. The range of variation of the friction coefficient *μ*_*i*_ was taken to be between 0 and 0.2 (this extreme value may correspond to steel/steel contact without any lubrification). In view of these results, the maximum *C*_*i*_/*C*_rg_ ratio was obtained with *C*_ax/sa_/*C*_rg_ and the minimum one with *C*_sa/sh_/*C*_rg_. The friction torque ratios *C*_*i*_/*C*_rg_ progressed linearly with the friction coefficient *μ*_*i*_ (where *μ*_sh_ = *μ*_sa/sh_ = *μ*_pl/sh_), as predicted by the constitutive equations in Section 2. Note that the results obtained in this first approximation are likely to differ from those obtained by simulating nonlinear behaviour between *C*_*i*_/*C*_rg_ and *μ*_*i*_.

**Figure 12:**
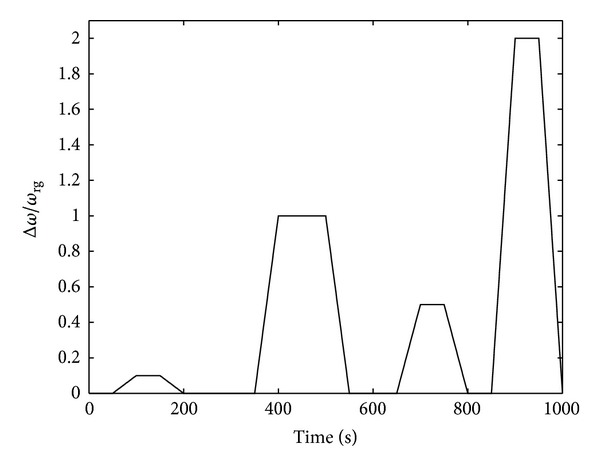
Example of a load parameter Δ*ω*/*ω*_rg_ with a complex path depending on time *t* ∈ [0,1000 s]: when Δ*ω*/*ω*_rg_ = 0 (in the case where *ω*_lw_ ≠ 0 and *ω*_rw_ ≠ 0) then *η*_di_ = 1 and *l*_*i*_ = *P*_*i*_/*P*_rg_ = 0, ∀*i* (when the vehicle was travelling along a straight line), when 0 < Δ*ω*/*ω*_rg_ < 2 then *η*_di_ < 1 and *l*_*i*_ = *P*_*i*_/*P*_rg_ > 0, ∀*i* (when the vehicle was taking a curvilinear course), when Δ*ω*/*ω*_rg_ = 2 then *η*_di_ < 1 and *l*_*i*_ = *P*_*i*_/*P*_rg_ > 0, ∀*i* (when one of the driven wheels underwent slipping, the vehicle was stationary); on the plateau, Δ*ω*/*ω*_rg_ = constant and |*ω*_lw_ − *ω*_rw_ | = constant (the vehicle was taking a constant curvilinear trajectory).

**Figure 13:**
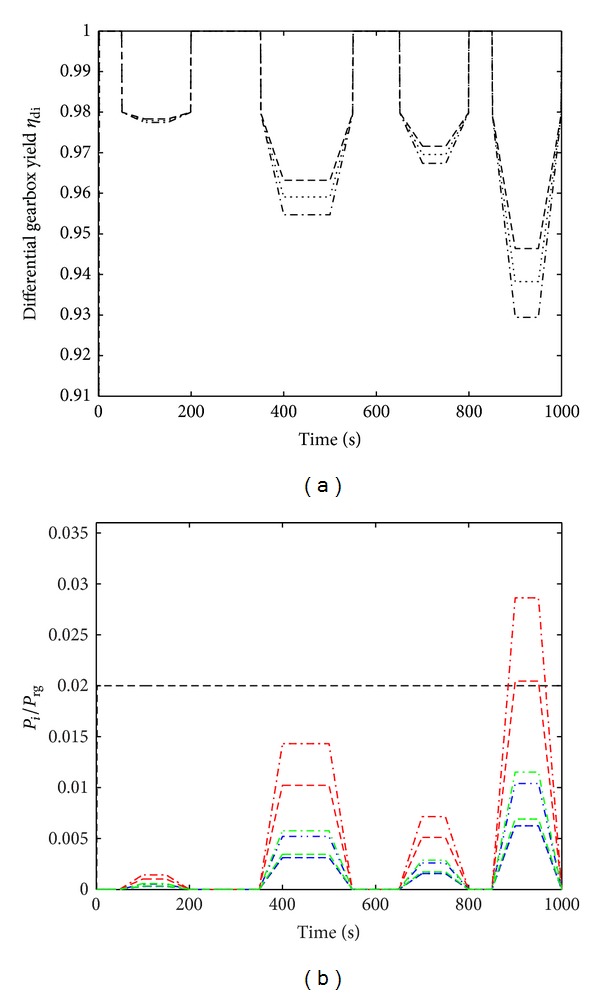
(a) Yield of a differential gearbox *η*_*di*_ with time *t* ∈ [0,1000 s] in the case of the complex path presented in Figure 12 with various sets of friction coefficients (*μ*_ax/sa_, *μ*_sh_): (0.03,0.05) in a dashed line, (0.05,0.07) in a dash-dot line, and (0.03,0.07) in a dotted line. (b) Loss ratio *l*_*i*_ = *P*_*i*_/*P*_rg_ of the *i*-mechanism (with *i* = (sa/pl, ax/sa, sa/sh, pl/sh)) with time *t* ∈ [0,1000 s] in the case of the complex path presented in Figure 12 with the same sets of friction coefficients as in (a): *l*_sa/pl_ = 0.96 which remained constant with time *t* (black solid line), *l*_ax/sa_ (red line), *l*_sa/sh_ (blue line), and *l*_pl/sh_ (green line).

A complex path of the load parameter Δ*ω*/*ω*_rg_ was then studied in order to test the ability of the model to describe the differential gearbox yield, *η*_di_, and the loss ratios *l*_*i*_ = *P*_*i*_/*P*_rg_ associated with the *i*-mechanism (where *i* = (sa/pl, ax/sa, sa/sh, pl/sh)) under more realistic conditions involving a straight line situation (Δ*ω*/*ω*_rg_ = 0 with *ω*_lw_ ≠ 0 and *ω*_rw_ ≠ 0), a curved line situation (0 < Δ*ω*/*ω*_rg_ < 2), a constant curved line situation (Δ*ω*/*ω*_rg_ = constant) or where one of the driven wheels undergoes slipping (Δ*ω*/*ω*_rg_ = 2).  Figure 12 shows the evolution of the load parameter Δ*ω*/*ω*_rg_ as a function of the time *t* ∈ [0,1000 s]. The results obtained with a complex path in terms of the yield of the differential gearbox (*η*_di_) and the loss ratios (*l*_*i*_) are shown in Figure 13. Specifically, Figure 13(a) gives the yield of the differential gearbox, *η*_di_, as a function of the time *t* ∈ [0,1000 s] in the case of the complex path plotted in Figure 12 with various sets of friction coefficients (*μ*_ax/sa_, *μ*_sh_): (0.03,0.05) in dashed line, (0.05,0.07) in dash-dot line, and (0.03,0.07) in dotted line. During the whole complex path of Δ*ω*/*ω*_rg_, the maximum (resp., minimum) yield of differential gearbox was reached with *μ*_ax/sa_ = 0.03 and *μ*_sh_ = 0.05 (resp., *μ*_ax/sa_ = 0.05 and *μ*_sh_ = 0.07). On the *k*-plate (with *k* = 1,2, 3,4) defined by the *k*-time interval [*t* ^*i*^, *t* ^*f*^]_*k*_, it can be noted that, for example (1) in [400 s, 500 s]_2_, *η*_di_ ≈ 0.9774 with (0.05,0.07), *η*_di_ ≈ 0.9591 with (0.03,0.07), and *η*_di_ ≈ 0.9547 with (0.05,0.07); (2) in [900 s, 950 s]_4_, *η*_di_ ≈ 0.9464 with (0.05,0.07), *η*_di_ ≈ 0.9382 with (0.03,0.07), and *η*_di_ ≈ 0.9294 with (0.05,0.07). The loss ratios due to friction *l*_*i*_ = *P*_*i*_/*P*_rg_ are shown in Figure 13(b). The maximum loss ratios obtained in decreasing order of magnitude (along the loading path) were *l*_sa/pl_ (black line), *l*_ax/sa_ (red line), *l*_pl/sh_ (green line), and *l*_sa/sh_ (blue line), except for the last load where *l*_ax/sa_ could be greater than *l*_sa/pl_ with the pair of friction coefficients (0.05,0.07) and (0.03,0.07). It should be noted that (1) on the second plate ([400 s, 500 s]_2_), *l*_ax/sa_^max⁡^ ≈ 0.0143 with (0.05,0.07), *l*_pl/sh_^max⁡^ ≈ 0.0057 with (0.03,0.07), and *η*_di_ ≈ 0.0052 with (0.05,0.07); (2) on the fourth plate ([900 s, 950 s]_4_), *l*_ax/sa_^max⁡^ ≈ 0.0286 with (0.05,0.07), *l*_pl/sh_^max⁡^ ≈ 0.0115 with (0.03,0.07), and *η*_di_ ≈ 0.0104 with (0.05,0.07).

Although only a sensitivity analysis was performed on the model, the results obtained show quite clearly that this model can be used to assess and predict the mechanical friction losses occurring in a differential gearbox. An experimental study shall be conducted in order to obtain more realistic values for some of the parameters, such as the friction coefficients (*μ*_ax/sa_ and *μ*_sh_), which significantly influence both the friction torques and the differential gearbox yield.

## 4. Conclusion

In this paper, an analytical model is presented for assessing and predicting the mechanical friction losses occurring in differential gearboxes. Some of the parameters involved in this model can be determined quite easily (geometric parameters), while others, which are more delicate, depend directly on the type of friction occurring in the mechanism (and therefore on the friction coefficients), which affects the mechanical losses to a variable extent. In order to test the influence of these parameters and determine the ability of the model to predict any mechanical losses, a sensitivity analysis was conducted. After this initial numerical approach, an experimental study shall be performed in order to obtain more realistic values for some of the parameters and confirm some of the assumptions made here in the modelling procedure.
